# Testosterone Exposure During Fetal Masculinization Programming Window Determines the Kidney Size in Adult Mice

**DOI:** 10.1096/fj.202500761RR

**Published:** 2026-04-04

**Authors:** Arttu Junnila, Kalle T. Rytkönen, Guillermo Martinez‐Nieto, Mats Perk, Otto Mäkelä, Hao Li, Jenni Airaksinen, Ida Hyötyläinen, Oliver Mehtovuori, Asta Laiho, Claes Ohlsson, Laura L. Elo, Satu Kuure, Matti Poutanen, Petra Sipilä

**Affiliations:** ^1^ Institute of Biomedicine, Research Centre for Integrative Physiology and Pharmacology University of Turku Turku Finland; ^2^ Turku Bioscience Centre University of Turku and Åbo Akademi University Turku Finland; ^3^ Stem Cells and Metabolism Research Program, Faculty of Medicine University of Helsinki Helsinki Finland; ^4^ Turku Center for Disease Modeling (TCDM), institute of Biomedicine University of Turku Turku Finland; ^5^ Department of Internal Medicine and Clinical Nutrition, Institute of Medicine, the Sahlgrenska Academy Gothenburg University Gothenburg Sweden; ^6^ GM‐Unit, Laboratory Animal Center, Helsinki Institute of Life Science University of Helsinki Helsinki Finland

**Keywords:** AR, HNF4A, IGFBP5, kidney growth, masculinization programming window, testosterone

## Abstract

Kidney size is sex‐dimorphic and regulated by androgens in adult humans and mice. However, the effects of developmental androgen deficiency on kidneys remain elusive. We hypothesized that androgens program future kidney growth during fetal development. Male mice lacking the main testosterone‐producing enzyme HSD17B3 had reduced testosterone at embryonic day 15.5, but the concentrations increased by E18.5, creating a short time window of androgen deficiency resulting in reduced kidney size in adult males. In male *Hsd17b3* ^*−/−*^ kidneys, nephron development was qualitatively normal, but the number of glomeruli and proliferation of proximal tubules were reduced, as was proximal tubule size in adults. Testosterone supplementation at E14.5–17.5 normalized the renal size in adult *Hsd17b3* ^*−/−*^ males. Our data suggest that androgen receptor and HNF4A jointly regulate IGFBP5, putatively influencing FOXO1 and mTOR signaling to promote male‐specific kidney growth in the fetal period. In conclusion, we have identified a novel developmental programming effect on male kidneys, where fetal androgen deficiency reduces kidney growth and androgen responsiveness in adult males.

## Graphical abstract

Kidney size is sex‐dimorphic and regulated by androgens in adult humans and mice. Male mice lacking the main testosterone‐producing enzyme HSD17B3 had reduced testosterone during a short fetal time window, leading to reduced size of kidneys in adults, which were not responsive to high adult testosterone. The androgen deficiency affected the signaling and early development of the proximal tubules. Fetal testosterone supplementation rescued the adult kidney size, revealing a novel developmental programming effect in kidneys.

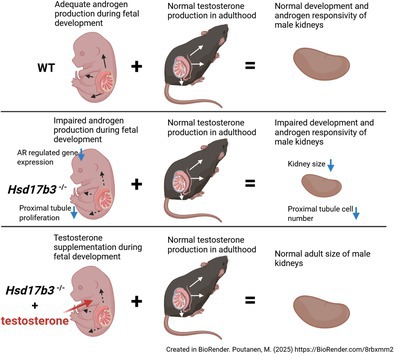

## 1 Introduction

Androgens regulate the development and function of not only reproductive organs, but also e.g., bone, adipose tissue, and kidney. In the kidney, androgens have long been known to regulate gene expression [1, 2], and recent scRNA‐seq analyses have further clarified the sex differences [3]. Differential expression of various Na^+^, K^+^, acid–base and organic acid transporters [4] in female and male kidneys contributes to sex differences in kidney functions, such as faster saline load excretion in female rats [5] and greater urinary ammonia excretion in female mice compared to males [6]. However, the physiological consequences of these sex differences for normal kidney function and in different diseases are largely unknown. Furthermore, male kidneys are larger in both rodents and humans [7]. Orchiectomy and testosterone replacement studies have demonstrated that adult kidney size is controlled by androgens [8, 9], and the effect is mediated via the androgen receptor (AR) located in the proximal tubule [10]. In newborn animals, kidney size is similar in females and males [11] and the size difference arises during puberty [9]. The androgen‐induced growth of the kidney in prepubertal/pubertal mice involves cell proliferation first and then cell hypertrophy of proximal tubule cells, and androgens are required to maintain cell size in the adult males [9]. In human kidney transplantation patients, kidney size 3 months after transplantation depended on the recipient sex, with males having larger tubular area [9], suggesting that androgens control kidney size also in humans.

We have previously generated a knock‐out mouse model for Hydroxysteroid (17β) dehydrogenase 3 (HSD17B3), which is the primary enzyme converting low‐active androgen androstenedione (A‐dione) to highly active testosterone (T) [12]. The *Hsd17b3* ^−/−^ male mice are undermasculinized, with reduced anogenital distance, delayed puberty, and subfertility. They have a similar endocrine imbalance to that of human patients with HSD17B3 deficiency, namely significant testosterone production but a low T/A‐dione ratio. Furthermore, the weights of several androgen‐sensitive tissues are reduced, including the kidney, regardless of high T levels in adult mice [13].

Development of male reproductive organs is dependent on fetal testosterone production, which starts around embryonic day 12.5–13 (E12.5–13) in mice and reaches a peak around E17–18 before declining again at birth [14]. Inhibition of androgen action during fetal development has been shown to affect the development of the reproductive tract [15, 16]. Further work with rats demonstrated a short masculinization programming window at E15.5 to E17.5, which determines the development of the male reproductive tract. Blockage of androgen action only from E15.5 to E17.5 caused underdeveloped internal genitalia, reduced length of phallus and anogenital distance, as well as increased number of hypospadias and cryptorchidism [17, 18]. Thus, androgens “preprogram” masculinization before actual morphological changes are observed in the developing organs.

Previous work demonstrating the androgen control of kidney growth has been done in post‐natal animals. Thus, the role of embryonic T exposure in kidney growth is unknown. In this work, we show that androgen action during the masculinization programming window also dictates the size of the kidney in adult mice. The reduced T levels at E15.5 embryos lead to significantly smaller kidneys in male mice, regardless of the high T levels in adult animals. Testosterone treatment of pregnant dams during the masculinization programming window at E14.5 to E17.5 rescued the kidney size to normal in male mice. We further identified signaling pathways putatively mediating the androgen effects in the developing kidney, linking decreased expression of *Igfbp5* to AR and *Hnf4a* regulation and further likely affecting *Mtor* and *Foxo1* levels.

## 2 Materials and Methods

### 2.1 Mouse Model

The generation and genotyping of the *Hsd17b3* ^−/−^ mouse line has been described by us previously [13]. Mice were housed under a controlled environment (12 h light cycle, temperature 21°C ± 3°C, humidity 55% ± 15%, specific pathogen‐free) at the Central Animal Laboratory of the University of Turku. Soy‐free SDS‐RM3 chow (Special Diets Service) and tap water were available ad libitum. All animal experiments were approved by the Finnish Animal Ethics Committee (licenses no. ESAVI/7487/04.10.07/2013, ESAVI/41729/2019, and ESAVI/23322/2023) and fully met the requirements as defined by the U.S. National Institutes of Health guidelines on animal experimentation.

### 2.2 Measurements, Body Composition, and Tissue Sampling

The anogenital distance was determined by measuring the distance between the external genitalia and the anus with a digital caliper (Hogetex). The measurements were done from neonatal animals and at the age of 3 months (WT *n* = 7, *Hsd17b3* ^−/−^ *n* = 8). The body weight of the animals was recorded at the age of 3 months. Body composition (fat mass and lean mass) was measured with the EchoMRI‐700 device (Echo Medical Systems, Houston, TX, USA) in live animals at the age of 3 months (WT *n* = 18, *Hsd17b3* ^−/−^ *n* = 20).

For the collection of tissues, mice were sacrificed at predetermined ages by carbon dioxide asphyxiation, followed by blood collection via cardiac puncture and cervical dislocation. Kidneys were weighed and snap‐frozen in liquid nitrogen or fixed for histological analyses. For samples from fetal time points, pregnant dams were sacrificed after timed matings at 15.5 days or 18.5 days post coitum (dpc) and kidneys and testes were collected from embryos.

### 2.3 Testosterone Rescue Experiments

On 14.5–17.5 dpc, pregnant dams were treated with testosterone propionate (Sigma‐Aldrich) in corn oil (Sigma‐Aldrich), administered s.c. once daily at 20 mg/kg. Male *Hsd17b3* ^−/−^ (*n* = 8) and WT (*n* = 9) pups were left to mature and were sacrificed at the age of 3 months.

For fetal kidney RNAseq, pregnant dams were treated either with testosterone propionate or with corn oil as vehicle control and sacrificed at 16.5 dpc, 2 h after the third testosterone propionate injection. The embryos (vehicle‐treated WT *n* = 4, vehicle‐treated *Hsd17b3* ^−/−^ *n* = 6, T‐treated WT *n* = 4, T‐treated *Hsd17b3* ^−/−^ *n* = 6) were collected, and the kidneys were snap‐frozen in liquid nitrogen for RNA extraction.

### 2.4 Steroid Analyses

The testes were homogenized in sterile deionized water 1:10 (w/v) using an Ultra‐Turrax homogenizer (IKA‐Werke). The concentrations of androstenedione and testosterone in the testes of E15.5 (WT *n* = 12, *Hsd17b3* ^−/−^ *n* = 10) and E18.5 (WT *n* = 13, *Hsd17b3* ^−/−^ *n* = 15) fetuses were analyzed by a validated GC–MS/MS, with the quantification limits of 12 pg/mL and 8 pg/mL respectively, as previously described [19].

### 2.5 Histological Analyses

Kidneys from 3‐month‐old mice were fixed in 10% neutral buffered formalin (FF‐Chemicals) for approximately 24 h at RT. The samples were then dehydrated, embedded in paraffin, 5 μm sections were prepared, deparaffinized, and rehydrated in a xylene‐ethanol series, stained with hematoxylin and eosin (HE), and analyzed via light microscopy.

### 2.6 Immunohistochemistry and Immunofluorescence Staining of Tissue Sections

The localization of the androgen receptor in WT mouse kidneys at E15.5 and 3 months of age was visualized with immunohistochemistry. Antibodies are listed in Table 1. The antigen retrieval for tissue sections was performed in a pressure cooker in 10 mM citrate buffer. Blocking was done with 3% bovine serum albumin (BSA) in PBST (PBS with 0.05% Tween). The primary antibodies were diluted in the blocking solution and incubated at 4°C overnight. Endogenous peroxidase activity was blocked with a 20‐min wash in 1% H_2_O_2_. The color formation was achieved with Dako EnVision HRP Labeled Polymer Anti‐Rabbit and Dako liquid DAB+ (K4003; Agilent) followed by a brief background staining with Mayer's hematoxylin and mounting. The results were analyzed via light microscopy or scanned using the Pannoramic P1000 slide scanner (3DHistech). For the analysis of proximal tubule size and cell number at 3 months of age, the cross‐sectional area of the proximal tubules, identified by AR staining and excluding lumen, was determined using the SlideViewer software (3DHistech). The cell number was determined as the number of AR‐stained nuclei in the cross‐section, and the cell size as the area divided by cell number. 15 randomly selected round and even tubular cross‐sections were measured and counted from 4 WT and 4 *Hsd17b3* ^−/−^ kidney sections.

**TABLE 1:** Primary and secondary antibodies and their use.

Antibody	Species	Manufacturer	Cat #	RRID	Dilution	Used in
anti‐AR antibody	Rabbit	Abcam	ab133273	AB_11156085	1:500	IH
anti‐Cyclin D1	Rabbit	Thermo Scientific	RM‐9104‐S1	AB_149913	1:500	IF
anti‐Jagged 1	Rat	Hybridoma Bank	TS1‐15H	AB_528317	1:300	IF
anti‐LEF1	Rabbit	Cell Signaling Technology	2230	AB_823558	1:400	IF
anti‐PAX2	Rabbit	Invitrogen	71–6000	AB_2533990	1:400	IF
anti‐Calbindin	Goat	Santa Cruz Biotechnology	sc‐7691	AB_634520	1:500	IF
anti‐Podocalyxin	Goat	R&D Systems	AF1556	AB_354858	1:400	IF
anti‐HNF4A	Mouse	Invitrogen	MA1‐199	AB_2633309	1:50	IF
anti‐Phosphohistone H3	Mouse	Cell Signaling Technology	9706	AB_331748	1:200	IF
anti‐Lrp2	Rabbit	Abcam	ab76969	AB_10673466	1:1000	IF
anti‐IGFBP5	Goat	R&D Systems	AF578	AB_2123485	1:500	IF, WB
anti‐rabbit IgG AlexaFluor 594	Goat	Life Technologies	A11037	AB_2534095	1:1000	IF
anti‐rabbit IgG AlexaFluor 488	Donkey	Invitrogen	A‐21206	AB_2535792	1:1000	IF
anti‐mouse IgG AlexaFluor 488	Goat	Life Technologies	A11029	AB_2534088	1:1000	IF
anti‐Goat IgG AlexaFluor 594	Donkey	Invitrogen	A‐11058	AB_142540	1:1000	IF
anti‐AKT	Rabbit	Cell Signaling Technology	4691	AB_915783	1:1000	WB
anti‐Phospho‐AKT (ser473)	Rabbit	Cell Signaling Technology	9271	AB_329825	1:1000	WB
anti‐Phospho‐S6 ribosomal protein (Ser235/236)	Mouse	Cell Signaling Technology	2211	AB_331679	1:1000	WB
Anti‐Beta‐Actin	Mouse	Sigma‐Aldrich	A1978	AB_476692	1:2500	WB
Anti‐rabbit IgG, HRP‐linked	Goat	Cell Signaling Technology	7074	AB_2099233	1:5000	WB
Anti‐mouse IgG, HRP‐linked	Horse	Cell Signaling Technology	7076	AB_330924	1:5000	WB

Abbreviations: IH, immunohistochemistry; IF, immunofluorescence; WB, western blot.

For immunofluorescence, the developmental stages of nephrons in E16.5 fetal kidneys were stained with antibodies for CYCLIN D1, JAGGED 1, LEF1, and PAX2, alongside CALBINDIN antibody as an epithelial marker as previously reported [20]. For counting the glomeruli in 2‐week kidneys, the kidneys were sectioned through (WT *n* = 2, 32 sections; *Hsd17b3* ^−/−^ *n* = 3, 62 sections), and podocytes were stained with PODOCALYXIN antibody. HNF4A staining was performed to E18.5 (WT *n* = 4, *Hsd17b3* ^−/−^ *n* = 3) and 3‐month‐old (WT *n* = 3, *Hsd17b3* ^−/−^ *n* = 3) kidneys. Cell proliferation in the proximal tubule epithelium of E18.5 and 2‐week kidneys was analyzed by staining mitosis marker phospho‐HISTONE H3 and LRP2 as a proximal tubule marker. The stainings were quantified using 19 WT sections and 13 *Hsd17b3* ^−/−^ sections at E18.5, and 12 WT sections and 12 *Hsd17b3* ^−/−^ sections at 2 weeks, and normalized to LRP2‐stained area. From the same 2 week stainings, relative proximal tubule area was quantified from representative cortical region images (2 per animal) based on the LRP2 staining using the ImageJ (version 1.52a) software [21]. Expression of IGFBP5 in proximal tubule was also confirmed with staining alongside LRP2 at E18.5 and 2 weeks. Antibodies are listed in Table 1.

Antigen retrieval was performed in a pressure cooker in Tris–HCl EDTA buffer (E18.5 kidneys) or citrate buffer (2‐week kidneys). Blocking and primary antibody incubation were carried out as above, followed by a 40‐min incubation with secondary antibodies. Finally, a brief incubation with DAPI was followed by mounting and imaging the slides with Zeiss Axio Imager (Carl Zeiss NTS Ltd.) or scanning them with Pannoramic MIDI fluorescent slide scanner (3DHistech).

### 2.7 Gene Expression Analyses and RNA Sequencing

Total RNA was extracted from E18.5 kidneys (WT *n* = 4, *Hsd17b3* ^−/−^ *n* = 5) as well as WT E15.5, 0 day and 3‐week‐old kidneys (*n* = 3 for all) with Trisure (Bioline) following the manufacturer's instructions. The RNA integrity of the samples was confirmed with NanoDrop ND‐1000 spectrophotometer (Thermo Fisher Scientific). One μg of RNA was treated with DNase Amplification Grade Kit (Thermo Fisher Scientific) and used for cDNA synthesis (SensiFast, Bioline). The cDNA was used to quantify gene expression by qPCR (CFX96 Real‐Time PCR detection system, Bio‐Rad) with the DyNAmo Flash SYBR Green qPCR Kit (Thermo Fisher Scientific). Gene expression was analyzed with primers for mouse *Hnf1b, Hnf4a*, and *Igfbp5*. Data was normalized to the expression of housekeeping genes *L19* and *Ppia*, and expression relative to the WT levels (Hnfs) or lowest expressing time point (*Igfbp5*) was calculated from Ct values using the ΔΔCt method [22]. Primers are listed Table 2.

**TABLE 2:** Polymerase chain reaction (PCR) primer sequences.

Gene	Primer name	Primer sequence
*Hnf1b*	mHnf1 Fw	CCGCATCCCAGCAAATTTTG
*Hnf1b*	mHnf1 Re	CCAGTTGTAGACACGGACCT
*Hnf4a*	mHnf4a Fw	ATCTCTGGGATCAATGGCGA
*Hnf4a*	mHnf4a Re	GGAGCAGCACGTCCTTAAAC
*Igfpb5*	mIgfbp5 Fw	CCGAGATGGCTGAAGAGACC
*Igfpb5*	mIgfbp5 Re	TCACAGTTGGGCAGGTACAC
*L19*	L19 Fw	GGACAGAGTCTTGATGATCTC
*L19*	L19 Re	CTGAAGGTCAAAGGGAATGTG
*Ppia*	Ppia Fw	CATCCTAAAGCATACAGGTCCTG
*Ppia*	Ppia Re	TCCATGGCTTCCACAATGTT

For RNA sequencing, total RNA was extracted from E16.5 kidneys from the testosterone supplementation experiment as described above. The quality of the RNA samples was confirmed by Bioanalyzer. Library preparation and sequencing were performed by Novogene Co. with the Illumina NovaSeq 6000 system. Computational analyses are described separately below.

### 2.8 Cell Culture and Proliferation

For proliferation analysis, HEK 293 cell line was obtained from ATCC and tested negative for mycoplasma contamination. Cells were cultured on poly‐L‐lysine–coated plates (#P4832, Sigma‐Aldrich) and switched to serum‐free medium (DMEM/F12, #D2906, Sigma; 1% Penicillin/streptomycin, #A5256701, Gibco; 1% L‐Glutamine, #25030–024, Gibco; 20 nM DHT) for 3 h before changing to fresh serum‐free medium with 25, 50, or 100 nM recombinant IGFBP5 (#578‐B5, R&D Systems). Cells were cultured for 24 h, and the cell proliferation was analyzed with an MTT cell proliferation and cytotoxicity assay kit (#E‐CK‐A341, Elabscience), measured on an EnSight microplate reader (Perkin Elmer).

### 2.9 Immunoblotting

For Western blotting, E18.5 KO and WT kidneys were homogenized in RIPA buffer with phosphatase inhibitor, and the proteins were separated on Mini‐ProteanTGX 4%–20% gels (#456–1094, BioRad) and transferred to PVDF membrane with BioRad SemiDry system at 25 V for 30 min. Membranes were blocked in a solution of 3% BSA and 5% fat‐free milk. Immunoblotting was performed with antibodies for AKT, Phospho‐AKT (pAKT), phospho‐S6, IGFBP5, and β‐actin overnight. Secondary staining was performed for 1 h in RT with HRP‐conjugated antibodies, and chemiluminescence detection reagents (#NEL122001EA, PerkinElmer) were used for imaging with Fujifilm LAS 4000 gel imager. Signal intensities were quantified with ImageJ software [21], normalized to β‐actin values as a loading control. Antibodies are listed in Table 1.

### 2.10 Computational Analyses

The analyses of AR chromatin binding upon testosterone activation in the kidney were performed utilizing previously published ChIP‐seq data (GSM1146473, GSM1146474, GSM1146475, GSM1259359 and GSM1259358 from GSE47192) [23] and UCSC Genome Browser (mm9) [24] or IGV viewer [25].

RNA‐seq data analysis was performed using a combination of programs. The quality of the raw sequencing reads was checked with FastQC tool version 0.11.14 [26]. Further analyses were carried out using R version 3.6.1 and Bioconductor version 3.9 [27]. The reads were aligned to the UCSC mm10 mouse genome reference, derived from Illumina iGenomes (https://support.illumina.com/sequencing/sequencing_software/igenome.html), using Rsubread package (version 2.0.0) [28] and its inbuilt Refseq gene annotation. Rsubread was also used to calculate the genewise read counts. Normalization was performed using the calcNormFactors function from the edgeR package version 3.28.0 [29], implementing the trimmed mean of M‐values (TMM) normalization method. Statistical testing between sample groups was carried out using ROTS package version 1.14.0 [30], and the differentially expressed genes were selected, requiring an unadjusted P‐value below 0.05. The volcano plot was generated with the VolcaNoseR web app [31].

Gene ontology (GO) enrichment analysis was performed using a threshold‐free gene‐set enrichment (GSEA) approach with gage package (version 2.36.0) [32]. The range of genes per term required was set between 15 and 250, and the test was performed with comparison scheme “as.group” using the function “gs.KSTest” for the non‐parametric Kolmogorov–Smirnov test. The gene lists sorted based on average ranks of both statistical significance and fold‐change from the differential expression testing were used as input.

Gene expression data were also analyzed using QIAGEN Ingenuity Pathway Analysis (IPA) [33]. Canonical pathway analysis identified the pathways from QIAGEN IPA library of canonical pathways that were most significant to the data set. Only genes from the dataset that met a P‐value cutoff of 0.05 and were associated with a canonical pathway in the QIAGEN Knowledge Base were considered. The significance of the association between the data set and the canonical pathway was measured using a right‐tailed Fisher's Exact Test.

The data set containing gene identifiers and their corresponding expression values was uploaded into the QIAGEN IPA software, and networks were generated. Each identifier was mapped to its corresponding entity molecule in QIAGEN's Knowledge Base. These molecules were overlaid onto a global molecular network developed from information contained in QIAGEN's Knowledge Base. Networks of eligible molecules were then algorithmically generated based on their connectivity, and selected networks were merged and combined to understand the underlying molecular mechanisms.

### 2.11 Statistical Analyses

Statistical analyses were done using GraphPad Prism 10 software (GraphPad Software). The normality of the data was evaluated based on a Shapiro–Wilk normality test. Statistical difference between two groups was determined by two‐tailed Student's *t*‐test or Mann–Whitney test for normally and non‐normally distributed data, respectively. For comparison of multiple groups, one‐way ANOVA and Tukey's multiple comparisons test, or Kruskal‐Wallis test and Dunn's multiple comparisons test were used for normally and non‐normally distributed data, respectively. Significance was determined as *p* ≤ 0.05, and results are shown as individual values, with a centerline indicating the mean, unless stated otherwise. All experiments presented in the Article were repeated in at least three independent biological replicates. No statistical methods were used to predetermine sample sizes. Data collection and analysis were not performed blinded. No other data points were removed from the experiments.

## 3 Results

### 3.1 Kidney Size is Reduced in Adult *Hsd17b3* ^−/−^ Male Mice

**FIGURE 1:**
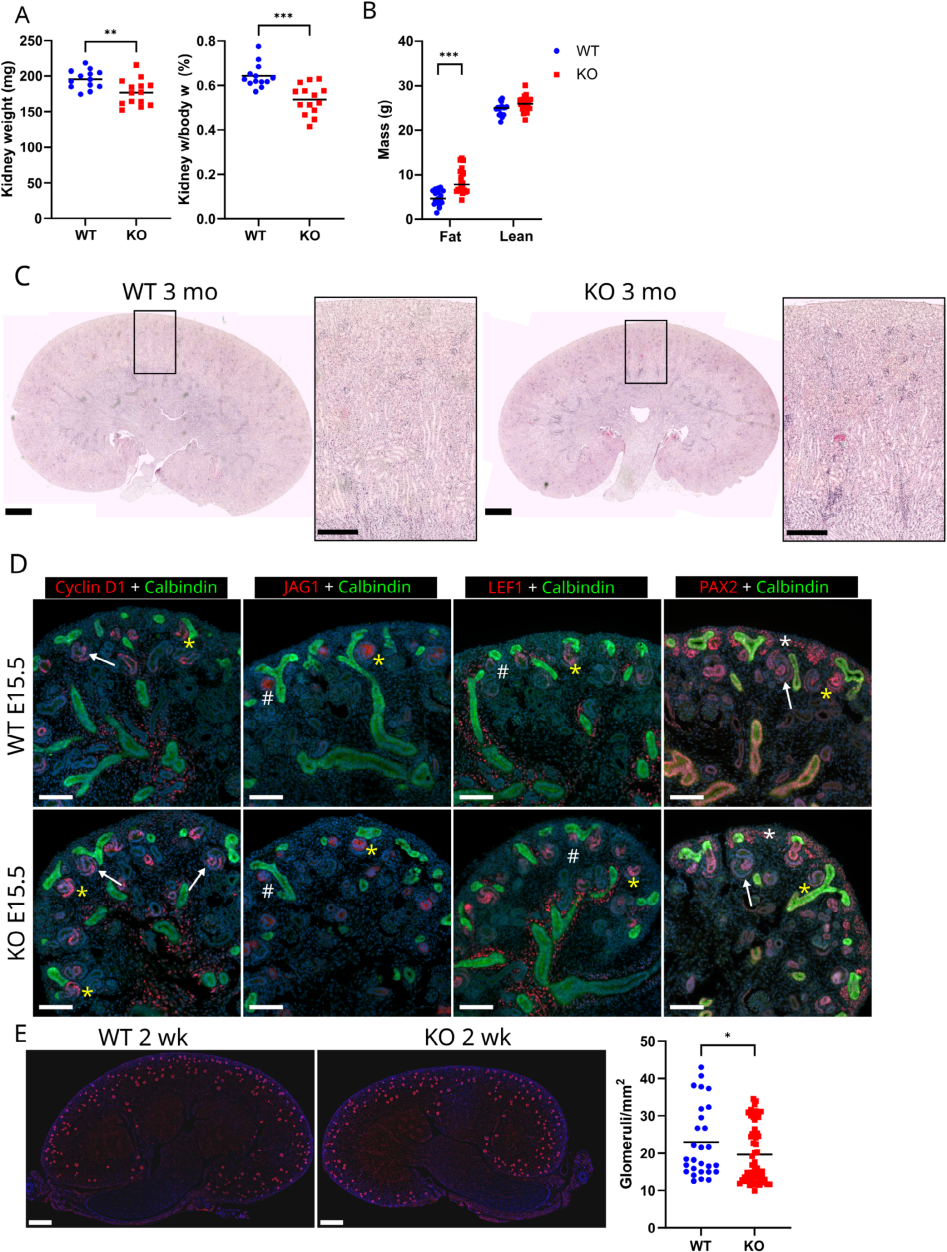
The kidneys of adult *Hsd17b3* ^−/−^ male mice are reduced in size but morphologically normal. (A) The weight of *Hsd17b3* ^−/−^ (KO) male kidneys (*n* = 14) at the age of 3 months in comparison with WT controls (*n* = 13), as absolute weight and percentage of body weight. The lines indicate means. ** = *p* ≤ 0.01, *** = *p* ≤ 0.001. (B) The fat and lean masses of *Hsd17b3* ^−/−^ (KO) male mice (*n* = 20) at the age of 3 months in comparison with WT controls (*n* = 18). *** = *p* ≤ 0.001. (C) Representative histological images of HE stained WT and *Hsd17b3* ^−/−^ (KO) kidneys at the age of 3 months. Scale bars: 1000 μm in wider, 400 μm in closer field images. (D) Immunofluorescent stainings of Cyclin D1, JAG1, LEF1, and PAX2 alongside Calbindin in E15.5 WT and *Hsd17b3* ^−/−^ (KO) kidneys. Scale bars: 100 μm. Yellow asterisk = comma shaped body, white asterisk = nephron progenitor, arrow = S‐shaped body, hash mark = pretubular aggregate. (E) Representative image and quantification of glomeruli at 2 weeks of age via immunofluorescent staining of PODOCALYXIN, counted from 32 WT sections and 62 *Hsd17b3* ^−/−^ (KO) sections. Scale bar: 500 μm. The lines indicate means. * = *p* ≤ 0.05.

One of the androgen‐sensitive tissues affected by the lack of HSD17B3 is the kidney, despite the high level of circulating testosterone in adult males [13]. In the current study, the kidneys of 3‐month‐old *Hsd17b3* ^−/−^ male mice were confirmed to be approximately 10% smaller in weight than in WT controls (*p* ≤ 0.01) (Figure 1A). The difference in the kidney weight normalized to body weight between the genotypes was even greater (Figure 1A). This was mainly explained by the greater body weight of the *Hsd17b3* ^−/−^ males we have reported previously [13]. The body weight difference, however, was mainly due to excess fat mass, and thus likely separate from the effect on organ weight, as the lean mass was similar between groups (Figure 1B). Regardless of the smaller size, histological analysis revealed no morphological abnormalities in the *Hsd17b3* ^−/−^ kidneys (Figure 1C).

Furthermore, the fetal development of nephron segments and overall kidney patterning appeared normal. We performed immunofluorescent staining of E15.5 kidneys with nephron precursor markers Cyclin D1 (pretubular aggregate to S‐shaped body), Jagged 1 (distal renal vesicle, comma‐ and S‐shaped bodies), LEF1 (pretubular aggregate, distal renal vesicle and distal comma‐ and S‐shaped bodies), and PAX2 (nephron progenitors and all nephron structures) along with ureteric bud epithelium marker Calbindin (Figure 1D). These studies revealed no impairment in the induction or patterning of the future nephrons. However, counting the number of glomeruli in 2‐week‐old male kidneys by staining PODOCALYXIN revealed a 15% decrease in the number of glomeruli per section area in *Hsd17b3* ^−/−^ kidney (*p* ≤ 0.05) (Figure 1E).

### 3.2 The Lack of HSD17B3 Leads to a Delay in Fetal Androgen Production

**FIGURE 2:**
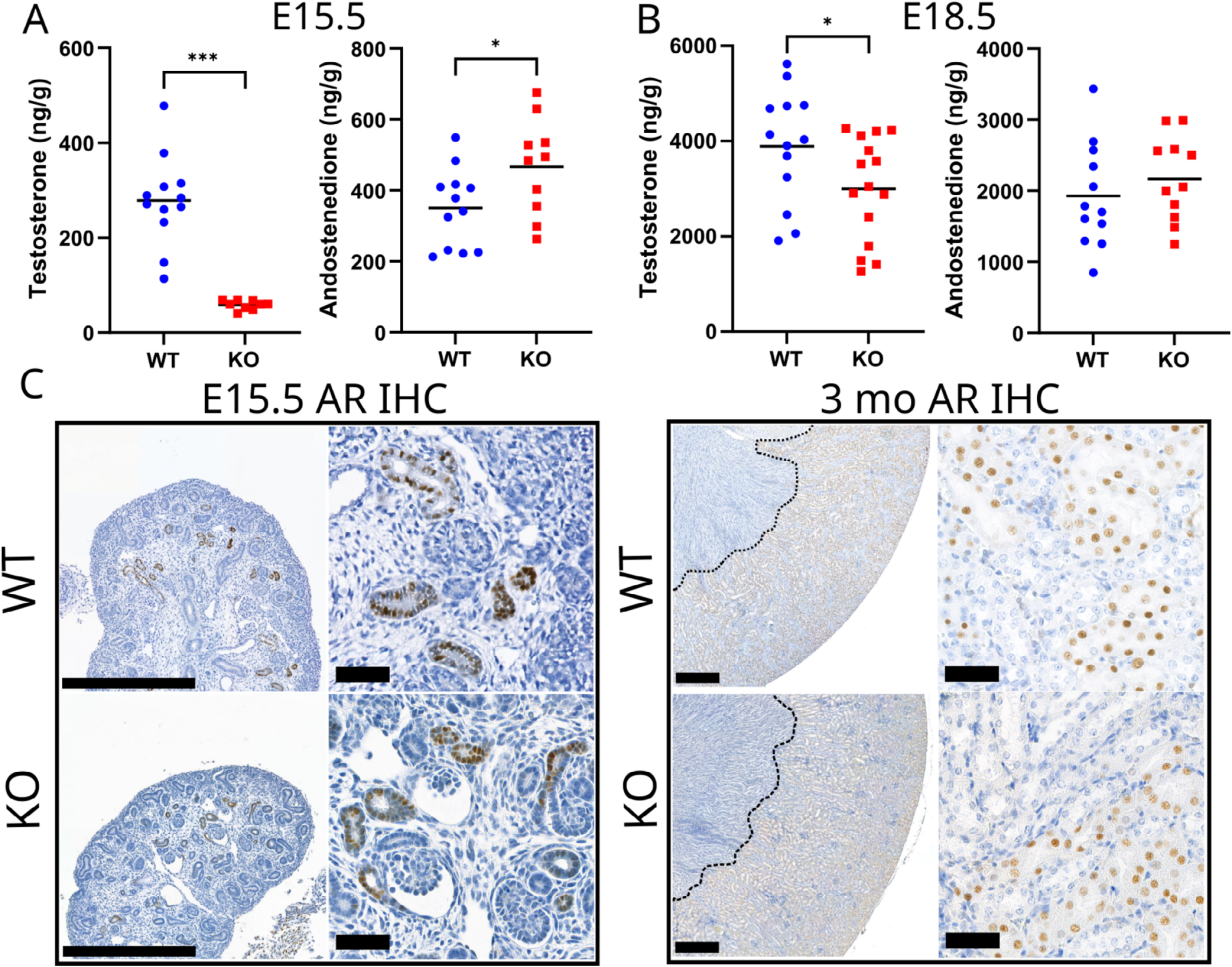
A fetal androgen deficiency in *Hsd17b3* ^−/−^ has an effect on androgen‐regulated genes and cell proliferation in the kidney. (A) Intratesticular testosterone and androstenedione concentrations in WT and *Hsd17b3* ^−/−^ (KO) at embryonal day 15.5 (E15.5). The lines indicate means. * = *p* ≤ 0.05 *** = *p* ≤ 0.001. (B) Intratesticular testosterone and androstenedione concentrations in WT and *Hsd17b3* ^−/−^ (KO) at E18.5. The lines indicate means. * = *p* ≤ 0.05. (C) Immunohistochemical staining of AR expression in E15.5 and 3‐month‐old WT and *Hsd17b3* ^−/−^ (KO) kidneys. Dotted line delineates the stained area in wider field images. Scale bars on wider field images represent 500 μm, on zoomed images 50 μm.

Although the *Hsd17b3* ^−/−^ mice were previously seen to have an undermasculinized phenotype, the circulating testosterone concentrations were normal or increased at the 27‐day or 3‐month time points, respectively [13]. Therefore, we suspected that the phenotype resulted from an earlier androgen deficiency during fetal development. Indeed, an apparent 5‐fold reduction in intratesticular testosterone concentration was observed at E15.5 (*p* ≤ 0.001), with a corresponding 1.3‐fold rise in androstenedione concentration (*p* ≤ 0.05) (Figure 2A). However, already by E18.5 the intratesticular testosterone concentration had risen close to normal, and androstenedione was at normal levels (Figure 2B). The lack of HSD17B3 activity therefore led to a delay in fetal testosterone production. We have previously shown that by birth, compensatory mechanisms elevate testosterone production in *Hsd17b3* ^−/−^ to normal level [34]. Thus, androgen deficiency was only observed during a short period in late fetal development, coinciding with the masculinization programming window from E15.5 to E17.5 identified for the reproductive organs.

### 3.3 Fetal Androgen Deficiency Affects Gene Expression and Cell Proliferation in the AR‐Expressing Proximal Tubule

Expression of AR was observed similarly in WT and *Hsd17b3* ^−/−^ male kidneys both during fetal development at E15.5 and in adults at 3 months of age (Figure 2C). At both time points, the expression localized solely in the proximal tubule segment of the nephron, the only region with known sex differences in gene expression [3].

**FIGURE 3:**
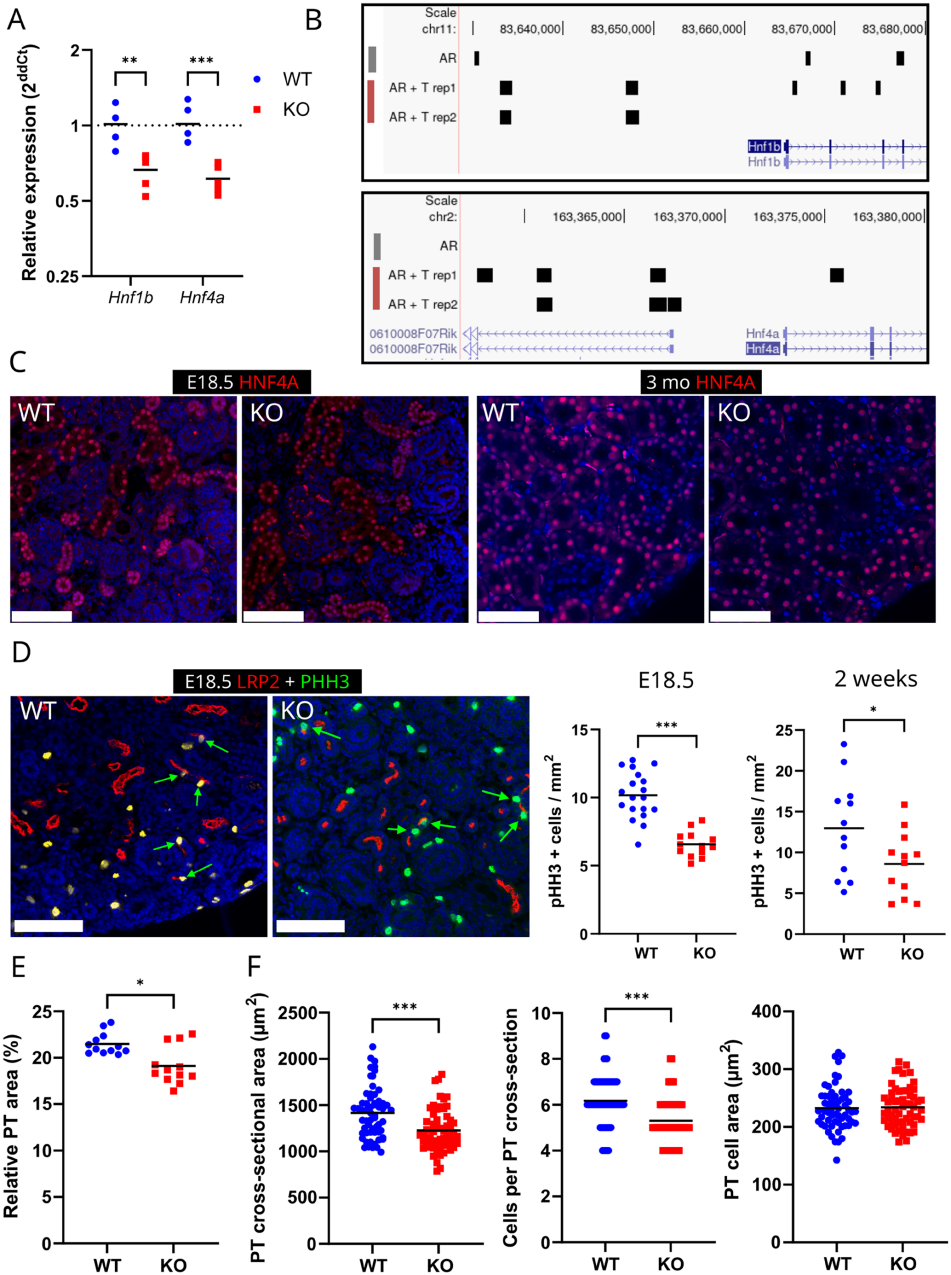
The fetal androgen deficiency affects the gene expression and growth of the proximal tubule. (A) RT‐qPCR analysis of *Hnf1b* and *Hnf4a* mRNA levels in E18.5 WT and *Hsd17b3* ^−/−^ (KO) kidneys. The lines indicate means. ** = *p* ≤ 0.01 *** = *p* ≤ 0.001. (B) Visualization of AR binding peaks near *Hnf1b* and *Hnf4a* in AR ChIP‐seq data from Pihlajamaa et al., 2014. (C) Immunofluorescent staining of HNF4A in E18.5 and 3‐month‐old WT and *Hsd17b3* ^−/−^ (KO) kidneys. Scale bar: 100 μm. (D) Representative image (E18.5) and quantification of cell proliferation in E18.5 (19 WT sections from 4 animals and 13 *Hsd17b3* ^−/−^ (KO) sections from 3 animals) and 2‐week‐old (12 WT sections from 6 animals and 12 *Hsd17b3* ^−/−^ sections from 6 animals) kidneys by immunofluorescent stainings of mitosis marker pHH3 (green) and proximal tubule marker LPR2 (red). Green arrows indicate pHH3‐positive cells in proximal tubule epithelium. The lines indicate means. Scale bar: 100 μm. * = *p* ≤ 0.05. (E) Quantification of LPR2‐stained proximal tubule area as a percentage in representative images of cortex area from the same sections (12 images from 6 WT animals and 12 images from 6 *Hsd17b3* ^−/−^ animals) from 2‐week‐old animals. The lines indicate means. * = *p* ≤ 0.05. (F) Quantification of proximal tubule cross‐sectional area, cell number and cell area in 3‐month‐old kidneys; 15 tubule cross‐sections each were counted from 4 WT sections and 4 *Hsd17b3* ^−/−^ (KO) sections. The lines indicate means. *** = *p* ≤ 0.001.

As hepatocyte nuclear factor family members *Hnf1b* and *Hnf4a* have been linked to the size determination of various tissues, including the kidney [35, 36], we next analyzed their expression in *Hsd17b3* ^*−/−*^ male kidneys. The mRNA levels of both genes were downregulated at E18.5, right after the period of androgen deficiency (Figure 3A). A re‐analysis of AR ChIP‐seq data from a study by Pihlajamaa et al. [23] showed AR peaks both at the promoter and intronic regions of both *Hnf1b* and *Hnf4a* (Figure 3B), suggesting that they are likely directly regulated by AR. Their study also identified *Hnf4a* as a pioneer factor in mouse kidney AR signaling, and the expression of *Hnf4a* was responsive to testosterone treatment at 12 h timepoint in adult castrated mice (fold change 1.52, *p* ≤ 0.02) [23]. To determine whether the lower mRNA expression of *Hnf4a* in whole kidney was due to lower AR‐regulated expression in the proximal tubule cells, or a reduced number of proximal tubules, we performed immunofluorescent stainings of E18.5 and 3‐month‐old kidneys, and detected comparable HNF4A expression in the proximal tubules in both genotypes (Figure 3C). We also identified a number of other proximal tubule marker genes (*Gsta2*, *Cyp2e1*, and *Sord* as markers of the whole proximal tubule; *Apoe* and *Slc5a2* for S1 segment; *Slc7a13* and *Kap* for S2 segment; and *Slc22a13* for S3 segment) in a kidney cell‐type specific RNAseq database [37, 38]. We analyzed their expression in our RNAseq data from E16.5 time point to identify wider changes in the expression profile, possibly indicating a lower number of developing proximal tubule structures, but none of the analyzed markers showed significant differences between WT and *Hsd17b3* ^*−/−*^, although most had a slight tendency towards being lowered in the latter (Table 3). Neither was there a difference in additional markers for other nephron segments (distal tubule marker *Slc12a3* and podocyte marker *Nphs2*).

**TABLE 3:** Expression of proximal tubule marker genes in RNAseq data.

Gene	WT vehicle	Hsd17b3^−/−^ vehicle
*Gsta2*	43.8 ± 16.9	37.4 ± 13.9
*Cyp2e1*	2.6 ± 1.6	1.6 ± 1.2
*Sord*	28.8 ± 5.2	24.0 ± 4.6
*Apoe*	107.1 ± 10.1	119.8 ± 19.5
*Slc5a2*	7.0 ± 2.6	5.5 ± 1.2
*Slc7a13*	13.2 ± 2.7	11.0 ± 3.3
*Kap*	14.8 ± 12.9	6.2 ± 10.1
*Slc22a13*	9.4 ± 3.0	7.6 ± 2.6
*Slc12a3*	18.7 ± 5.4	15.8 ± 3.9
*Nphs2*	29.0 ± 6.4	27.1 ± 4.8

*Note:* No significant differences were observed between groups for any analyzed marker. Vehicle‐treated WT *n* = 3, vehicle‐treated *Hsd17b3* ^−/−^ *n* = 6. Values are Reads Per Kilobase per Million mapped reads (RPKM), presented as means ±SD.

In order to more directly identify the possible changes in the growth of proximal tubules, we analyzed cell proliferation at E18.5 and in the rapidly growing kidneys of 2‐week‐old males by double IF staining of low‐density lipoprotein receptor‐related protein 2 (LRP2) as a marker of proximal tubule segments, and phospho‐HISTONE H3 (pHH3) as a proliferation marker. The analysis demonstrated that the number of proliferating cells within the proximal tubule epithelium was significantly smaller in both E18.5 (*p* ≤ 0.001) and 2‐week‐old (*p* ≤ 0.05) *Hsd17b3* ^−/−^ kidneys compared to WT (Figure 3D). As a result, the relative area of proximal tubules in the cortex was also reduced in *Hsd17b3* ^−/−^ kidneys at 2 weeks of age (Figure 3E). The reduced cell proliferation indicates that fetal androgen deficiency does have a persisting effect on the growth of the androgen‐sensitive proximal tubules both immediately and during later development. As the effect of androgens on proximal tubule growth has previously been shown to involve both epithelial hypertrophy and hyperplasia, we aimed to quantify the effect in adult kidneys by determining the average proximal tubule cross‐sectional area, cell number, and cell size in 3‐month‐old mice. While the average cell size was unchanged, the number of cells and thus the tubule cross‐section was seen to be significantly reduced in *Hsd17b3* ^−/−^ kidneys (Figure 3F).

### 3.4 Fetal Testosterone Supplementation Rescues the Adult Kidney Phenotype

**FIGURE 4:**
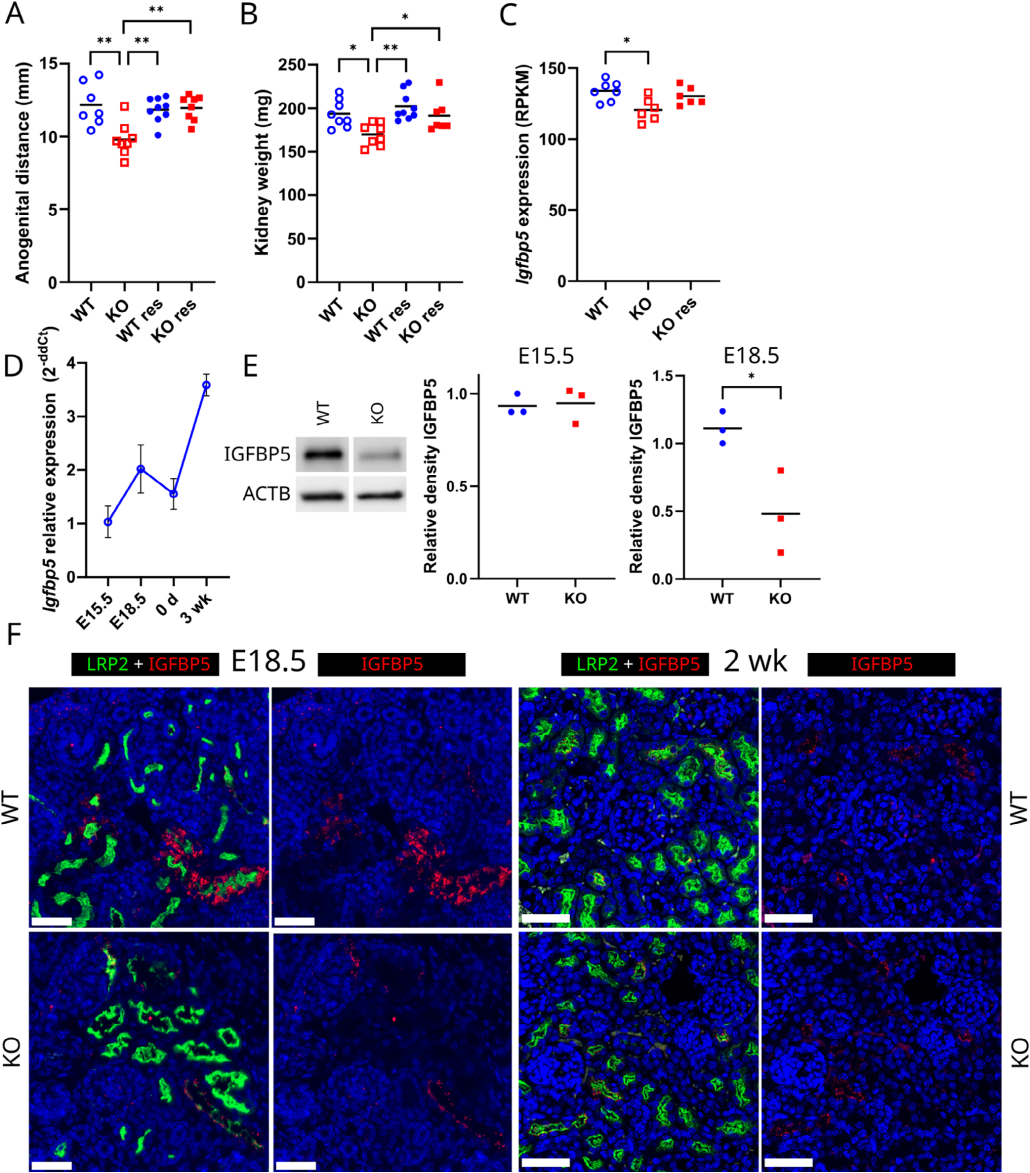
Fetal testosterone supplementation rescues the adult kidney size by normalizing gene expression in the fetal kidney. (A) Anogenital distance of 3‐month‐old *Hsd17b3* ^−/−^ (KO) and WT males without and with testosterone supplementation (res) at E14.5–17.5. The lines indicate means. * = *p* ≤ 0.05 ** = *p* ≤ 0.01. (B) kidney weights of 3‐month‐old *Hsd17b3* ^−/−^ (KO) and WT males without and with testosterone supplementation (res) at E14.5–17.5. The lines indicate means. * = *p* ≤ 0.05 ** = *p* ≤ 0.01. (C) *Igfbp5* expression (RNA‐seq) in E16.5 kidneys of WT, *Hsd17b3* ^−/−^ (KO) and treated KO (res) groups (RPKM = Reads Per Kilobase Million). The lines indicate means. * = *p* ≤ 0.05. (D) *Igfbp5* mRNA expression (qPCR) at different age points in WT kidneys. Points indicate means and whiskers SD. d = day, wk. = week. (E) Representative western blot images in E18.5 and quantification of IGFBP5 and loading control β‐actin in E15.5 and E18.5 WT and *Hsd17b3* ^−/−^ (KO) kidney homogenates. The lines indicate means. * = *p* ≤ 0.05. (F) Immunofluorescent staining of IGFBP5 (red) and LRP2 (green) in E18.5 and 2‐week‐old WT and *Hsd17b3* ^−/−^ (KO) kidneys. Scale bar: 50 μm.

To test the effect of fetal androgen deficiency in the development of the kidney, we performed a testosterone treatment of pregnant dams during E14.5–17.5 and analyzed the adult phenotype of the resulting male pups. The fetal testosterone supplementation fully rescued the reduced anogenital distance of *Hsd17b3* ^−/−^ males at the age of 3 months (Figure 4A). Interestingly, the androgen supplementation also normalized the adult *Hsd17b3* ^−/−^ kidney weight, as a significant difference in weight was observed between testosterone‐treated *Hsd17b3* ^−/−^ and untreated *Hsd17b3* ^−/−^ (*p* ≤ 0.05), but no difference between treated *Hsd17b3* ^−/−^ and WT (Figure 4B). The result demonstrates that fetal androgen exposure does have a role in programming mouse kidney development.

To identify genes and pathways affected by fetal androgen deficiency and rescued by testosterone supplementation, we performed RNA‐seq analyses of testosterone‐treated and untreated kidneys of WT and *Hsd17b3* ^−/−^ male pups at E16.5. Using a more stringent cutoff (FDR < 0.05) did not identify any differentially expressed (DE) genes between the groups, likely reflecting the moderate quantitative phenotype. Therefore, we proceeded with exploratory analyses using the unadjusted P‐values (< 0.05) to identify candidate DE genes and transcriptional trends (Figure S1A). Gene ontology enrichment analysis of the 416 genes identified with these parameters to be differentially expressed between vehicle treated *Hsd17b3* ^−/−^ and WT kidneys revealed several likely affected biological processes relevant to the observed phenotype, including those involved in e.g., steroid metabolic process (FDR = 0.0047), regulation of tube diameter (FDR = 0.0047) and tube size (FDR = 0.0050) (Table 4).

**TABLE 4:** Top 15 gene ontology terms enriched in comparison of vehicle treated WT and *Hsd17b3* ^−/−^ fetal male kidneys.

GO ID	Description	FDR
GO:0015893	drug transport	0.00032
GO:0008202	steroid metabolic process	0.00472
GO:0008203	cholesterol metabolic process	0.00472
GO:0009581	detection of external stimulus	0.00472
GO:0009582	detection of abiotic stimulus	0.00472
GO:0035296	regulation of tube diameter	0.00472
GO:0042730	fibrinolysis	0.00472
GO:0050880	regulation of blood vessel size	0.00472
GO:0051606	detection of stimulus	0.00472
GO:0097746	regulation of blood vessel diameter	0.00472
GO:0016125	sterol metabolic process	0.00506
GO:0035150	regulation of tube size	0.00506
GO:1902652	secondary alcohol metabolic process	0.00506
GO:0050982	detection of mechanical stimulus	0.01208
GO:0097756	negative regulation of blood vessel diameter	0.01365

Abbreviation: FDR = false discovery rate.

### 3.5 Androgen Deficiency Downregulates the Expression of *Igfbp5*, Impacting Cell Proliferation and Signaling

**FIGURE 5:**
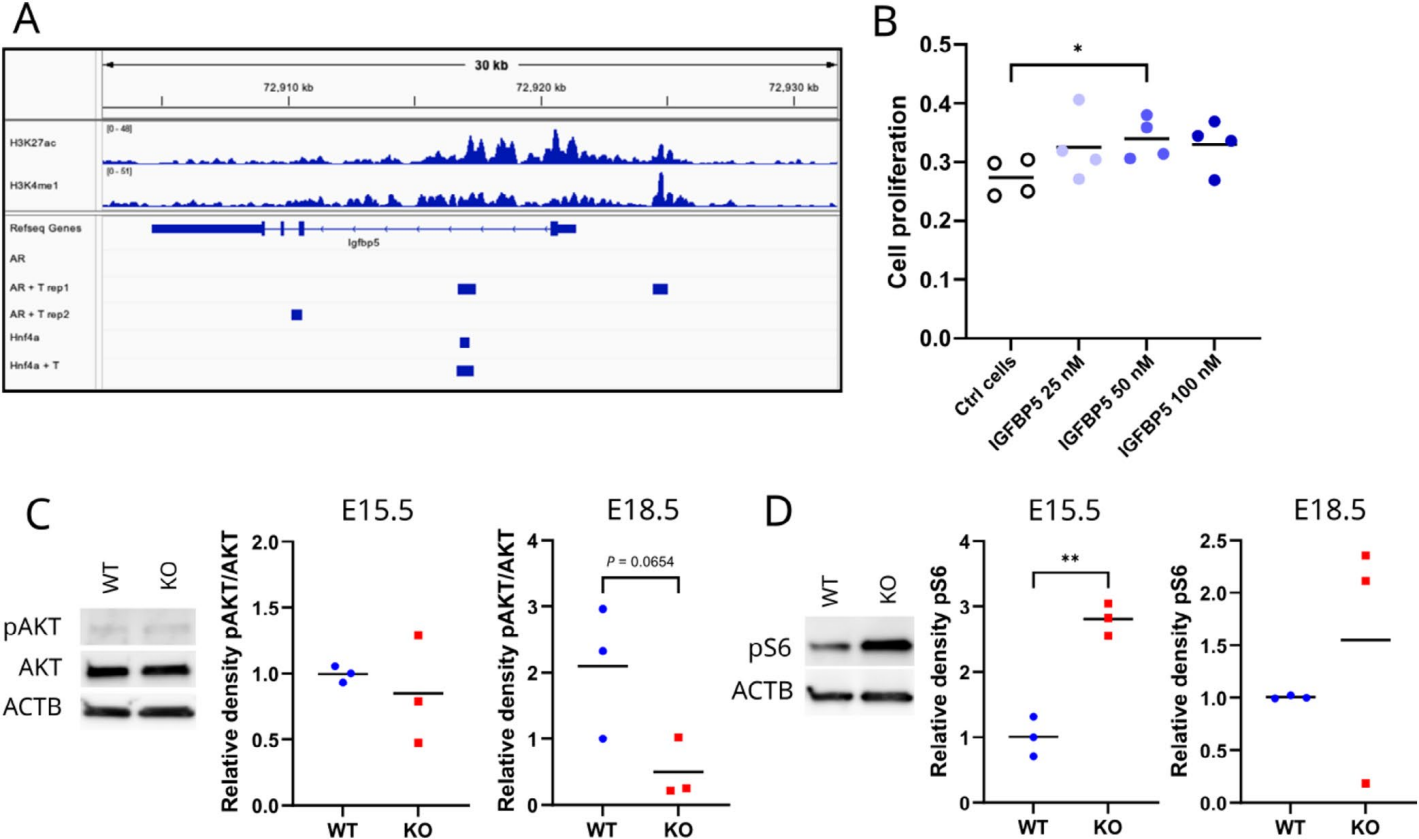
IGFBP5 is likely regulated by AR and HNF4A, and in turn can affect cell proliferation. (A) Visualization of AR and HNF4A binding peaks and H3K4m1 and H3K27ac histone marks near *Igfbp5* in ChIP‐seq data from Pihlajamaa et al., 2014. T = testosterone treatment. (B) Proliferation analysis of HEK 239 cells cultured with 0 (Ctrl), 25, 50, or 100 nM recombinant human IGFBP5. The lines indicate means. * = *p* ≤ 0.05. (C) Representative western blot images in E15.5 and quantification of phosphorylated AKT (pAKT), AKT, and loading control β‐actin in E15.5 and E18.5 WT and *Hsd17b3* ^−/−^ (KO) kidney homogenates. The lines indicate means. (D) Representative western blot images in E15.5 and quantification of phosphorylated S6 ribosomal protein (pS6) and loading control β‐actin in E15.5 and E18.5 WT and *Hsd17b3* ^−/−^ (KO) kidney homogenates. The lines indicate means. ** = *p* ≤ 0.01.

By comparing the candidate DE genes between untreated WT and *Hsd17b3* ^−/−^ kidneys, but not DE between untreated WT and testosterone‐treated *Hsd17b3* ^−/−^ kidneys, we identified 292 genes whose expression in *Hsd17b3* ^−/−^ was normalized by the testosterone supplementation (Table S1), leading to normalized kidney weight. We compared this set of genes with previously published scRNA‐seq datasets from newborn or adult kidneys [3, 39, 40] to identify genes co‐expressed with AR in the proximal tubules, as possible direct targets of androgen regulation. From the 292 DE genes in *Hsd17b3* ^−/−^ kidneys, 11 were found to be expressed in the proximal segment of nephrons (Table S1). One of those, insulin‐like growth factor binding protein 5 (*Igfbp5*, Figure 4C), caught our attention as a secretory factor that could mediate the effects of androgen action from proximal tubules to other areas of the kidney. In WT kidneys, *Igfbp5* mRNA expression increased by E18.5, then decreased slightly by birth, and rose again at 3 weeks of age, aligning with changes in androgen production during development (Figure 4D). The decrease of IGFBP5 protein expression in *Hsd17b3* ^−/−^ kidneys at E18.5 was confirmed in Western blot analysis (Figure 4E). The proximal tubule localization of IGFBP5 in E18.5 and 2‐week old *Hsd17b3* ^−/−^ and WT kidneys was affirmed by immunofluorescent staining, demonstrating staining in many, but not all, tubules that also stained with LRP2 (Figure 4F). Furthermore, reanalysis of an AR ChIP‐seq experiment [23] demonstrated AR binding sites in the promoter as well as first and second introns of *Igfbp5*, one of which also overlapped with an HNF4A binding site (Figure 5A). Additionally, histone modifications H3K27ac and H3K4me1 overlapped with AR and HNF4A binding sites suggesting open chromatin (Figure 5A). To establish whether IGFBP5 deficiency could lead to the observed growth defect in *Hsd17b3* ^−/−^ kidneys, we treated HEK 293 cells with IGFBP5 recombinant protein. The supplementation of HEK 293 cells with increasing concentrations of IGFBP5 significantly increased their proliferation, as shown by a 1.2‐fold increment with 50 nM IGFBP5 (*p* ≤ 0.05) (Figure 5B).

We then performed IPA pathway analysis from the 292 genes normalized in *Hsd17b3* ^−/−^ kidney by testosterone supplementation. The top ten pathways notably included the regulation of the eIF4 signaling pathway, the eIF2 signaling pathway, and the hepatic fibrosis signaling pathway (Figure S1 B). All these contained downregulated mechanistic target of rapamycin kinase (*Mtor*) gene (*p* ≤ 0.05), eukaryotic translation initiation factor 4E member 3 (*Eif4e3*, *p* ≤ 0.05), and forkhead box O1 (*Foxo1*, *p* ≤ 0.005). Furthermore, IPA network analysis linked *Igfbp5* with FOXO1 and mTOR signaling via AKT function (Figure S1 C). As AKT activation through phosphorylation is an essential component of these pathways, we analyzed the ratio of pAKT to total AKT in E15.5 and E18.5 kidneys with Western blotting. At E18.5, a lower portion of AKT was phosphorylated in *Hsd17b3* ^−/−^ kidneys compared to WT, although the difference did not reach statistical significance due to high variation in WT samples (Figure 5C). mTORC1 activation leads to phosphorylation of ribosomal protein S6, and thus we analyzed phosphoS6 from fetal kidneys. However, phosphoS6 was found to be significantly increased in E15.5 *Hsd17b3* ^−/−^ kidneys (Figure 5D).

## 4 Discussion

Kidney size in mice and humans is sensitive to androgen exposure, demonstrated by the rapid effect of androgen deprivation and reintroduction on tubule mass and total organ size through AR [9]. Therefore, it was not surprising that a mouse model lacking a crucial enzyme of testosterone production would have reduced kidney size. Comparable size reduction has been observed in males of a mouse model that has a mutation impairing AR dimerization [41], as well as in male mice with a kidney‐specific AR knockout^10^, suggesting that the effect seen in our study is AR dependent. However, unlike the lifelong disruption of androgen action in AR models, the adult *Hsd17b3* ^−/−^ male mice still produced high amounts of testosterone. This suggested a developmental mechanism regulating the growth potential of male kidneys independently of androgen exposure in adulthood.

In this study, we observed that the lack of HSD17B3 in male mice led to testosterone deficiency only during a short fetal time window. The sex difference in kidney size, however, has been shown to develop after puberty in response to androgen exposure and is not apparent during earlier development [9, 11]. It has previously been established that the growth potential of the male reproductive organs in rodents is determined by androgens during the fetal masculinization programming window [17, 18]. The target size is reached through later androgen exposure, but the final organ size will remain reduced if the early exposure is insufficient. We have now shown that a similar programming window exists for the mouse kidney: the smaller organ size in adult *Hsd17b3* ^−/−^ male mice results from a fetal testosterone deficiency. The high testosterone level, present in our model from puberty onward, is unable to later induce kidney growth to full male size [13], which is, however, rescued by fetal testosterone supplementation within the identified developmental time window. This is also supported by our previous results on another mouse model lacking both HSD17B1 and HSD17B3 activity. There we demonstrated a more complete abolishment of fetal testosterone production, resulting in an even greater reduction in adult kidney weight, yet the serum steroid profile after puberty was identical to *Hsd17b3* ^−/−^, including high testosterone [34]. Furthermore, even though there is previous evidence that neonatal androgen exposure or deprivation can have a similar programming effect on adult mouse kidney size and androgen responsiveness [11], our model demonstrates that even normal neonatal testosterone [34] is not sufficient if there is an earlier fetal deficiency.

The proximal tubule is the only structure of the kidney known to express AR and respond to androgens^3,9,10^. Here, too, we detected AR expression only in the cells of the proximal tubule, beginning from the fetal period. Moreover, significant down‐regulation of *Hnf1b* and *Hnf4a* mRNA levels was observed in E18.5 kidneys of *Hsd17b3* ^−/−^ males. As immunofluorescent staining didn't reveal any obvious differences in the HNF4A protein expression in the proximal tubules, it seems likely that the lower amount of detected mRNA is due to a general decrease in proximal tubule structures, which is congruent with the decreased proliferation detected at the same time point. However, both *Hnf1b and Hnf4a* have also been shown to be responsive to testosterone [23] and ChIP‐seq data showed AR binding to promoter and intronic regions of both genes. Furthermore, HNF4A has been shown to regulate differentiation of proximal tubule progenitor cells into mature proximal tubule cells [35, 42]. Thus, the detected down‐regulation of *Hnf4a* could also be partly due to the lack of androgens during preceding days in *Hsd17b3* ^−/−^ kidneys and, in turn, could putatively play a part in a reduction of the number of developing proximal tubule cells, leading to the defect in proliferation seen at E18.5. In any case, the early reduction in proliferation persists past the observed period of androgen deficiency. This early defect would explain why we observe a later reduction in proximal tubule size and cell number, despite both having previously been shown to be acutely responsive to high androgen levels even in adults [9]. As *Hnf4a* was recently predicted to regulate male‐biased transcriptome in the kidney, having considerable overlap in target genes with AR [43], and identified as a pioneer factor for AR in the kidney [23], the growth of male kidney would likely be mediated through their co‐operative control of gene expression in the male proximal tubule in response to androgens.

In our RNA‐seq analysis, a relatively small number of genes, 292, were putatively changed between the genotypes in E16.5 embryonic kidneys but normalized by testosterone supplementation. From those 292 candidate genes, only 11 were found to be expressed in the proximal tubules of newborn kidneys [3, 39, 40], making them possible targets of direct AR regulation. We focused on *Igfbp5*, which has been previously reported to be among castration‐responsive genes in the adult mouse kidney [23]. We demonstrated that the fetal downregulation was indeed detectable also at the protein level, and furthermore, that the gene has associated AR binding sites that also overlap with HNF4A binding, suggesting that IGFBP5 expression is one of the factors in male proximal tubule development co‐regulated by both AR and HNF4A [43].

IGFPB5 is a secreted factor that conveys its effects either via regulating IGF functions or independently of IGFs [44]. The lack of *Igfbp5* has been linked to e.g., increased body size in knock‐out studies, likely due to increased IGF‐1 activity in the animals [45]. On the other hand, IGFBP5 has been shown to increase the proliferation of papillary thyroid carcinoma cells [46] and mouse osteoblasts independently of IGF‐1 [47]. Here too, we demonstrated that the addition of recombinant IGFBP5 increased the proliferation of cultured human embryonic kidney (HEK 293) cells. Therefore, it is possible that it has a similar direct effect on the kidney in vivo.

Studies in human prostate cancer cell lines suggest that overexpression of IGFBP5 decreases PI3K and pAKT levels [48], and regardless of whether the effect is IGF‐1 dependent or independent, it would likely affect mTOR signaling. Our IPA network analysis linked downregulated *Igfbp5* with the presumptive downregulation of *Foxo1* and *Mtor* via predicted downregulation of AKT activation, and indeed, we observed a trend towards reduced pAKT levels in E18.5 kidney of *Hsd17b3* ^−/−^ males. IGFBP5 also contains a functional nuclear localization sequence [44], and a recent study with rat primary hypothalamic cells suggested that IGFBP5 increased the protein levels of mTOR and AKT [49]. Thus, the expression of *Foxo1* and *Mtor* could be affected by direct transcriptional regulation by IGFBP5. Moreover, *Mtor* itself has previously been identified as both a male proximal tubule‐specific and androgen‐regulated gene [3, 23]. Nevertheless, the androgen deficiency and downregulation of IGFBP5 did not reduce phosphoS6 levels downstream of mTOR in E15.5 and E18.5 kidneys, with a significant increase instead observed at E15.5. Our findings are, however, in line with previous studies; in adult male mice, orchiectomy increases phosphoS6 levels [9] similarly to T deficient E15.5 *Hsd17b3* ^−/−^ kidneys. Importantly, S6 phosphorylation can also be the result of e.g., the Ras–ERK pathway [50] and thus does not necessarily reflect the mTOR activity. On the other hand, lower *Mtor* expression has been shown to affect kidney size and reduce nephron number [51] which could explain decreased nephron number seen in our study. The mTOR pathway does not seem to have a role in the androgen responsive growth of postnatal proximal tubules, but postnatally involved Cyclin D1 or ornithine decarboxylase [9] in turn did not differ between *Hsd17b3* ^*−/−*^ and WT fetal kidneys in our transcriptomics data. This may reflect a difference between the signaling pathways mediating pre‐ and postnatal androgen‐induced proximal tubule growth, as the latter was seen to firstly involve increased proliferation, then increase in cell size [9]. No later impairment in cell size was detected in our analysis, perhaps due to sufficient postnatal testosterone exposure.

Our studies give new insights into human HSD17B3 deficiency, which causes a disorder of sex development in XY individuals. The results have relevance for patients whose endocrine phenotype is very similar: fetal androgen deficiency, followed by virilization after puberty as testosterone production is activated [13, 52]. There is evidence that a programming window for reproductive tissues may also exist in humans, theorized to be between 8 and 14 weeks of gestation, and adult mouse and human kidneys react to androgens in a very similar way [9, 17, 53]. Thus, a similar mechanism of kidney developmental programming in humans is plausible. Reduced kidney size comes with a diminished nephron number, which increases the risks of chronic kidney disease, hypertension and cardiovascular problems [54]. Importantly, the findings are relevant not only for individuals with HSD17B3 deficiency but also for other disorders such as 5α‐reductase deficiency, androgen insensitivity syndrome, or environmental exposure to hormonal disruptors. However, further work is needed to clarify the possible consequences of the changes in kidney development. The sex differences observed in the kidney likely have a physiological function. Sex‐atypical kidney phenotype may then affect its functional capacity, if not in normal conditions, then during infections, or challenges such as a high sodium diet or heavy use of e.g., certain painkillers [55, 56].

In conclusion, we showed that the lack of HSD17B3 and the resulting disruption of testosterone production leads to reduced kidney size in *Hsd17b3* ^−/−^ male mice. The origin of the effect is in a brief fetal time window of androgen deficiency at E15.5‐E18.5. This results in developmental changes that cannot be reversed by higher testosterone later in development, but are rescued by fetal testosterone supplementation. One likely mechanism behind the effect is a decrease in AR‐ and HNF4A‐regulated expression of IGFBP5 in the proximal tubule area, leading to decreased proliferative potential in the proximal tubules.

## Author Contributions

Conceptualization, S.K., M.P. and P.S.; formal analysis, A.J., K.R., G.M.‐N., M.P., A.L. and L.L.E.; investigation, A.J., O.M., H.L., J.A., I.H., and O.M.; resources C.O., S.K., M.P. and P.S.; Writing – Original Draft, A.J. and P.S.; Writing – Review and Editing, all authors; supervision C.O., L.L.E., S.K., M.P. and P.S.; Funding Acquisition, M.P. and P.S.

## Funding

Sigrid Juséliuksen Säätiö (Sigrid Jusélius Stiftelse), 10.13039/501100006306. Jalmari ja Rauha Ahokkaan Säätiö (Jalmari and Rauha Ahokas Foundation), 10.13039/100010125. Turun Yliopistosäätiö (Turku University Foundation), 10.13039/501100022793. Organon RD Finland.

## Conflicts of Interest

The authors declare no conflicts of interest.

## Supporting information


**Figure S1:** RNAseq and IPA gene interaction analysis.
(A) Volcano plot comparison of gene expression in E16.5 kidneys between WT and Hsd17b3‐/‐ males. Log_2_ fold change cut off −0.1–0.1, unadjusted *p*‐value cut off = 0.05.
(B) Top 10 most significant canonical pathways obtained from Ingenuity Pathway Analysis (IPA) of DE genes that were normalized in Hsd17b3‐/‐ kidney upon fetal androgen supplementation. The top x‐axis represents the ‐log (*P*‐value).
(C) Interaction network map. The network was generated by merging the individual networks of the molecules IGFBP5 and mTOR obtained after comparing differential gene expression between WT and Hsd17b3‐/‐. These factors were significantly represented in the top canonical pathway results.

**Table S1:** Genes differentially expressed between WT vehicle and KO vehicle, but not KO testosterone supplemented group.

## Data Availability

The RNA sequencing data has been deposited in the GEO database under accession number GSE280108. All the other data are presented in the manuscript or in the Supporting Information. Additional requests can be directed to the corresponding author.
