# Supplementary figures and images for: Testosterone Exposure During Fetal Masculinization Programming Window Determines the Kidney Size in Adult Mice
Source: FASEB J. 2026 Apr 4;40(7):e71724. doi: 10.1096/fj.202500761RR (PMC13050020; doi:10.1096/fj.202500761RR)

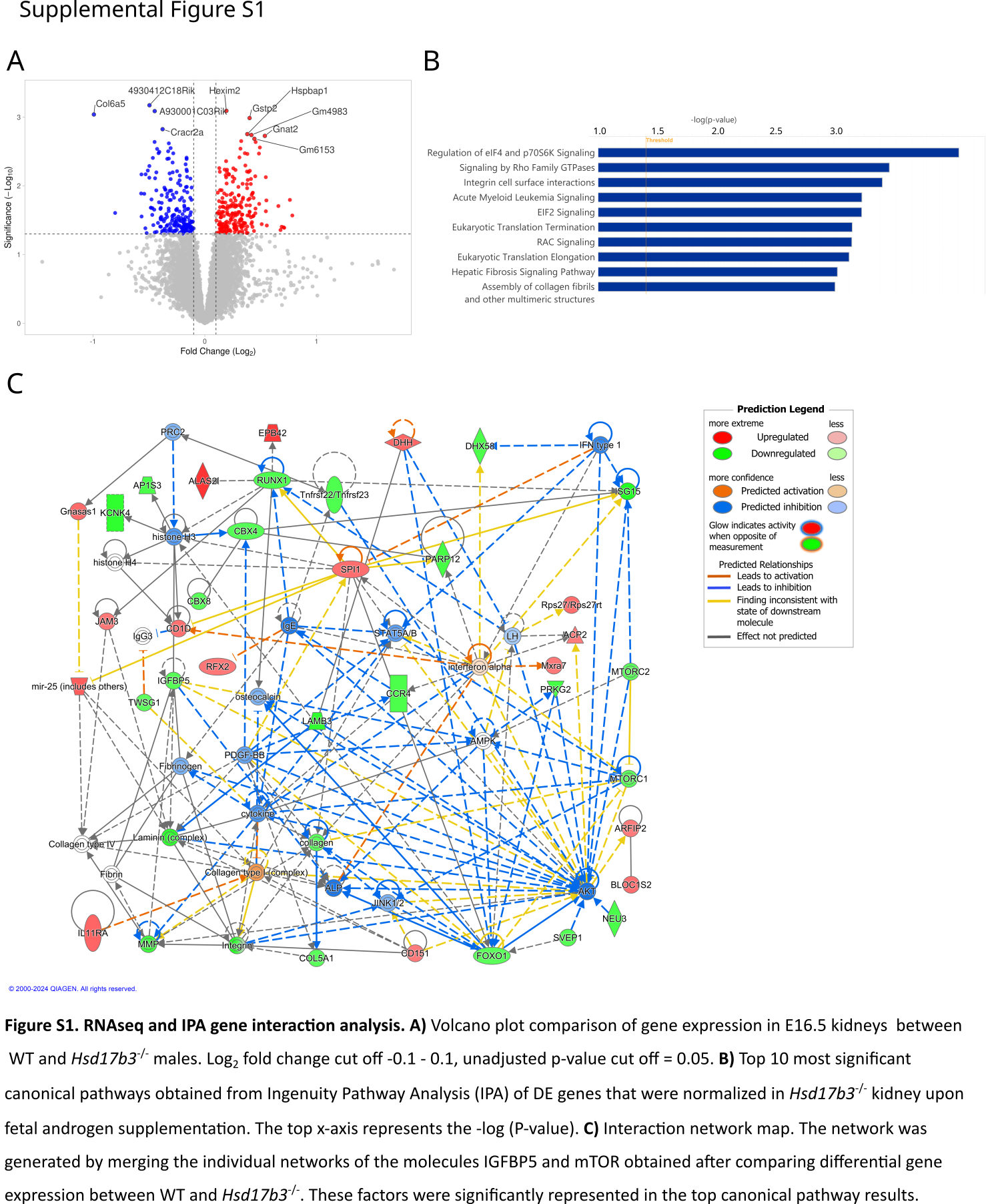

Supplement: Supplementary file 1 — Figure S1: RNAseq and IPA gene interaction analysis. (A) Volcano plot comparison of gene expression in E16.5 kidneys between WT and Hsd17b3‐/‐ males. Log2 fold change cut off −0.1–0.1, unadjusted p‐value cut off = 0.05. (B) Top 10 most significant canonical pathways obtained from Ingenuity Pathway Analysis (IPA) of DE genes that were normalized in Hsd17b3‐/‐ kidney upon fetal androgen supplementation. The top x‐axis represents the ‐log (P‐value). (C) Interaction network map. The network was generated by merging the individual networks of the molecules IGFBP5 and mTOR obtained after comparing differential gene expression between WT and Hsd17b3‐/‐. These factors were significantly represented in the top canonical pathway results. [file FSB2-40-e71724-s001.tif]
